# Pre-Quaternary divergence and subsequent radiation explain longitudinal patterns of genetic and morphological variation in the striped skink, *Heremites vittatus*

**DOI:** 10.1186/s12862-017-0969-0

**Published:** 2017-06-09

**Authors:** Felix Baier, Andreas Schmitz, Hedwig Sauer-Gürth, Michael Wink

**Affiliations:** 1 Department of Biology, Institute of Pharmacy and Molecular Biotechnology, Heidelberg University, Im Neuenheimer Feld 364, 69121 Heidelberg, Germany; 2 Natural History Museum of Geneva, Department of Herpetology & Ichthyology, route de Malagnou 1, 1208 Geneva, Switzerland; 3 Current address: Department of Organismic and Evolutionary Biology, Department of Molecular and Cellular Biology, Museum of Comparative Zoology, Harvard University, 16 Divinity Avenue, Cambridge, MA 02138 USA

**Keywords:** Phylogeography, Western Palearctic, Cline, Latitude, Longitude, Climate oscillation, *Heremites vittatus*, Intraspecific variation

## Abstract

**Background:**

Many animal and plant species in the Middle East and northern Africa have a predominantly longitudinal distribution, extending from Iran and Turkey along the eastern Mediterranean coast into northern Africa. These species are potentially characterized by longitudinal patterns of biological diversity, but little is known about the underlying biogeographic mechanisms and evolutionary timescales. We examined these questions in the striped skink, *Heremites vittatus*, one such species with a roughly longitudinal distribution across the Middle East and northern Africa, by analyzing range-wide patterns of mitochondrial DNA (mtDNA) sequence and multi-trait morphological variation.

**Results:**

The striped skink exhibits a basic longitudinal organization of mtDNA diversity, with three major mitochondrial lineages inhabiting northern Africa, the eastern Mediterranean coast, and Turkey/Iran. Remarkably, these lineages are of pre-Quaternary origin, and are characterized by *p*-distances of 9–10%. In addition, within each of these lineages a more recent Quaternary genetic diversification was observed, as evidenced by deep subclades and high haplotype diversity especially in the Turkish/Iranian and eastern Mediterranean lineages. Consistent with the genetic variation, our morphological analysis revealed that the majority of morphological traits show significant mean differences between specimens from northern Africa, the eastern Mediterranean coast, and Turkey/Iran, suggesting lineage-specific trait evolution. In addition, a subset of traits exhibits clinal variation along the eastern Mediterranean coast, potentially indicating selection gradients at the geographic transition from northern Africa to Anatolia. The existence of allopatric, morphologically and genetically divergent lineages suggests that *Heremites vittatus* might represent a complex with several taxa.

**Conclusions:**

Our work demonstrates that early divergence events in the Pliocene, likely driven by both climatic and geological factors, established the longitudinal patterns and distribution of *Heremites vittatus*. Subsequent radiation during the Pleistocene generated the genetic and morphological diversity observed today. Our study provides further evidence that longitudinal diversity patterns and species distributions in the Middle East and northern Africa were shaped by complex evolutionary processes, involving the region’s intricate geological history, climatic oscillations, and the presence of the Sahara.

**Electronic supplementary material:**

The online version of this article (doi:10.1186/s12862-017-0969-0) contains supplementary material, which is available to authorized users.

## Background

Intraspecific patterns of biological diversity are often the result of geological and climatic processes in the past. In the western Palearctic, the best understood example is the widespread presence of latitudinal gradients in genetic diversity in European taxa [1–4]. During interglacial periods in the Pleistocene, these species rapidly and repeatedly extended from Mediterranean refugia into central and northern Europe. Genetic bottlenecks during the cyclical expansions led to allele loss and decreased heterozygosity, and ultimately a reduction in genetic diversity in northern areas. While latitudinal gradients can be found in many species in Europe, the geographical shape of southern Europe made longitudinal movements more infrequent. Longitudinal patterns of genetic diversity are thus often confined to the different Mediterranean peninsulas, and can be difficult to disentangle from latitudinal gradients [5, 6].

Compared to continental Europe, relatively little is known about the structure and evolution of biological diversity in other regions of the Palearctic, notably in the Middle East and northern Africa [7–12]. Several species in northern Africa display post-glacial latitudinal range expansions, often into Europe despite the potential barrier of the Mediterranean Sea [13]. Remarkably, many species in this region share a roughly longitudinal distribution extending from Iran and Turkey along the eastern Mediterranean coast into northern Africa [6]. Unlike in Europe, strong sea barriers are absent in this region, which potentially facilitated longitudinal movements. In addition, the presence of the Sahara as a southern barrier for many species may have further supported longitudinal migration. European species with more pronounced longitudinal patterns of genetic diversity often originated in Asia Minor and expanded westwards along the north Mediterranean coast. As such, Asia Minor has become known as a center of genetic diversity in the Middle East [14–16]. However, whether species that expanded from Asia Minor into northern Africa instead of Europe are also characterized by longitudinal patterns remains poorly understood. More generally, few studies have addressed the biogeographic mechanisms and underlying evolutionary timescales in species with longitudinal distributions in this region [5, 6, 17, 18]. Here, we examined these questions in the striped skink, *Heremites vittatus*, a scincid lizard with a predominantly longitudinal distribution across the Middle East and northern Africa.

Lizards of the genus *Mabuya* sensu lato (s.l.) are some of the most widely distributed skinks with a circumtropical distribution. For a long time, these skinks were collectively allocated to the genus *Mabuya* s.l., until the Afro-Malagasy *Mabuya* taxa were assigned to the genus *Euprepis* [19, 20], and later re-assigned to *Trachylepis* [21]. The Middle Eastern *Mabuya* s.l. species were preliminarily included into *Trachylepis*, although they form a separate radiation [22]. To account for this, the genus *Heremites* was recently revalidated for these species, including *H. vittatus*, *H. auratus*, and *H. septemtaeniatus* [23]. Of these, *H. vittatus* has the largest distribution range, occurring in Algeria, Tunisia, Libya, Egypt, Israel, Jordan, Lebanon, Cyprus, Syria, Turkey, Iraq and Iran [24–29]. Despite its wide distribution, the striped skink has been regarded as a monotypic species [30]. In several parts of this large distribution range, e.g., in Turkey, Iran, and Lebanon, morphometric studies uncovered considerable morphological variation within local populations and differences between populations on a regional scale [31–36]. Part of this variation is likely due to adaptation to local environmental conditions, rather than phylogenetic divergence. For example, in a population in southeastern Turkey, uniform skinks are more abundant in habitats with low grass cover, while striped specimens are more abundant in habitats with high grass cover, indicative of disruptive selection by visual predators [36]. A distribution model for *H. vittatus* demonstrated that the species is predominantly found in areas with high winter precipitation (>300 mm), and rainy winters may be the driving factor behind its distribution [37]. Populations in northern Africa inhabit wetlands and oases [24], while those in Iran and Turkey live in mountainous areas [26]. Due to its poor dispersal skills and its dependence on humid habitats in the arid Middle Eastern environment, the striped skink is a sensitive indicator of geographic processes that have driven the distribution and intraspecific evolution of animals in this region [38].

In this work, we combined investigations of mtDNA (*cytochrome b*) sequence and multivariate morphological variation across the species’ distribution range, and provide the first comprehensive analysis of the biogeography and evolutionary history of the striped skink. We chose to study both genetic and morphological variation because evolutionary processes can affect genetics and morphology in different ways and at different times during the period of divergence. Unlike putatively neutral molecular markers, morphological traits are under varying degrees of selection; thus, it may be expected that morphological traits can show both clinal variation as a result of local adaptation, as well as trait conservation as a result of deep divergence events [39]. Together, insights from genetic and morphological variation can thus provide for a richer and more integrated understanding of the underlying evolutionary processes [39, 40].

## Results

### Sequence diversity

**Fig. 1:**
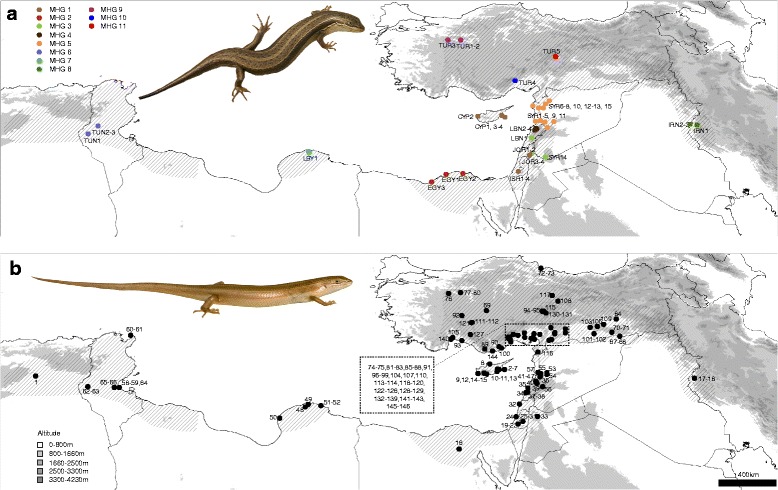
Distribution of *Heremites vittatus* and sample locations. **a** Samples included in the molecular phylogeography (see Additional file 3) and distribution of major haplogroups (MHG). For all figures, the color coding of MHGs is the same. **b** Samples included in the morphological analysis (Additional file 4). The distribution of *H. vittatus* is indicated by diagonal lines. Insets show the striped **a** and uniform **b** type of *Heremites vittatus* in the field near Chlorakas, Cyprus (photos by David J. Sparrow)

**Fig. 2:**
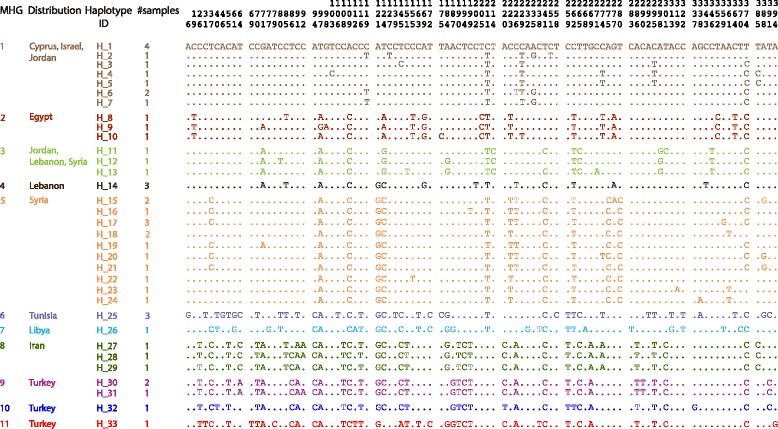
Variable sites among the 33 haplotypes in *Heremites vittatus*. Numbers at the top refer to the position of the variable site in the sequence alignment. A dot indicates an identical nucleotide compared to the reference sequence (CYP1)

**Fig. 3:**
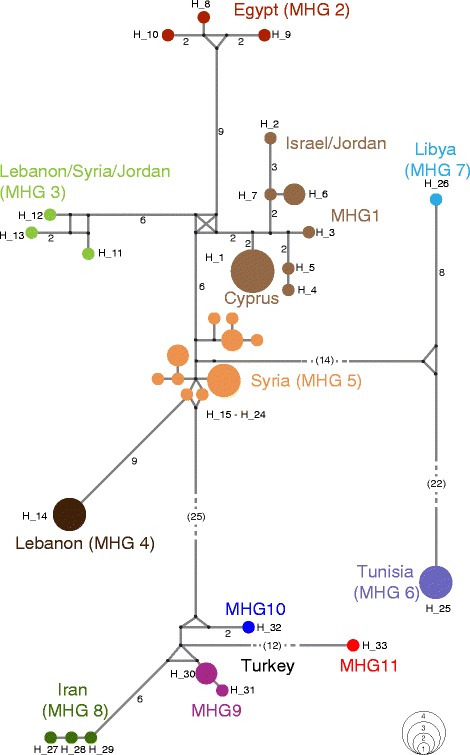
Haplotype network of *Heremites vittatus*. Numbers on branches represent nucleotide substitutions between haplotypes. The diameter of nodes is proportional to the number of samples with the respective haplotype (legend in bottom right)

Among the alignment of 394 nucleotides, 94 (24%) positions were variable and 78 (20%) were parsimony informative across the *Heremites vittatus* sequences. The 46 *H. vittatus* sequences comprised 33 individual haplotypes with a haplotype diversity of *H* = 0.9816, which we assigned to 11 major haplogroups (MHGs) (Figs. 1, 2; see Table 1 for *p*-distances between MHGs) based on the Bayesian PTP model (Additional file 1). The haplotype network contained three sub-networks, corresponding to haplotypes from (1) the populations in the eastern Mediterranean (MHGs 1–5), (2) Libya/Tunisia (MHGs 6–7), and (3) Turkey/Iran (MHGs 8–11) (Fig. 3).

**Table 1:** *P*-distances between major haplogroups. Below diagonal, mean *p*-distances between MHGs. Above diagonal, standard error of the mean (SEM) for mean *p*-distances between MHGs. On diagonal, mean *p*-distance ± SEM within MHGs. Standard errors were determined from 1000 Bootstrap replicates

	*H. auratus*	MHG1 (Cyprus, Israel, Jordan)	MHG2 (Egypt)	MHG3 (Jordan, Lebanon, Syria)	MHG4 (Lebanon)	MHG5 (Syria)	MHG6 (Tunisia)	MHG7 (Libya)	MHG8 (Iran)	MHG9 (Turkey)	MHG10 (Turkey)	MHG11 (Turkey)
*H. auratus*	0.062 ± 0.008	0.018	0.019	0.019	0.019	0.018	0.019	0.018	0.018	0.018	0.018	0.019
MHG1 (Cyprus, Israel, Jordan)	0.185	0.012 ± 0.003	0.009	0.008	0.009	0.008	0.014	0.013	0.013	0.013	0.014	0.014
MHG2 (Egypt)	0.196	0.044	0.010 ± 0.004	0.009	0.009	0.009	0.014	0.014	0.015	0.015	0.015	0.016
MHG3 (Jordan, Lebanon, Syria)	0.192	0.038	0.048	0.010 ± 0.004	0.009	0.008	0.014	0.013	0.013	0.013	0.013	0.014
MHG4 (Lebanon)	0.193	0.041	0.040	0.036	0.000	0.008	0.014	0.013	0.013	0.014	0.013	0.015
MHG5 (Syria)	0.184	0.038	0.044	0.039	0.029	0.010 ± 0.003	0.013	0.012	0.013	0.013	0.013	0.015
MHG6 (Tunisia)	0.200	0.102	0.103	0.095	0.094	0.094	0.000	0.013	0.015	0.015	0.015	0.016
MHG7 (Libya)	0.191	0.079	0.091	0.074	0.074	0.067	0.079	NA	0.015	0.014	0.014	0.015
MHG8 (Iran)	0.193	0.090	0.107	0.084	0.082	0.086	0.097	0.102	0.003 ± 0.002	0.007	0.008	0.010
MHG9 (Turkey)	0.193	0.089	0.108	0.085	0.085	0.084	0.105	0.100	0.023	0.002 ± 0.002	0.006	0.009
MHG10 (Turkey)	0.197	0.091	0.105	0.081	0.081	0.081	0.099	0.091	0.030	0.016	NA	0.010
MHG11 (Turkey)	0.194	0.101	0.123	0.096	0.102	0.101	0.112	0.096	0.041	0.036	0.041	NA

### Phylogeography and divergence times

**Fig. 4:**
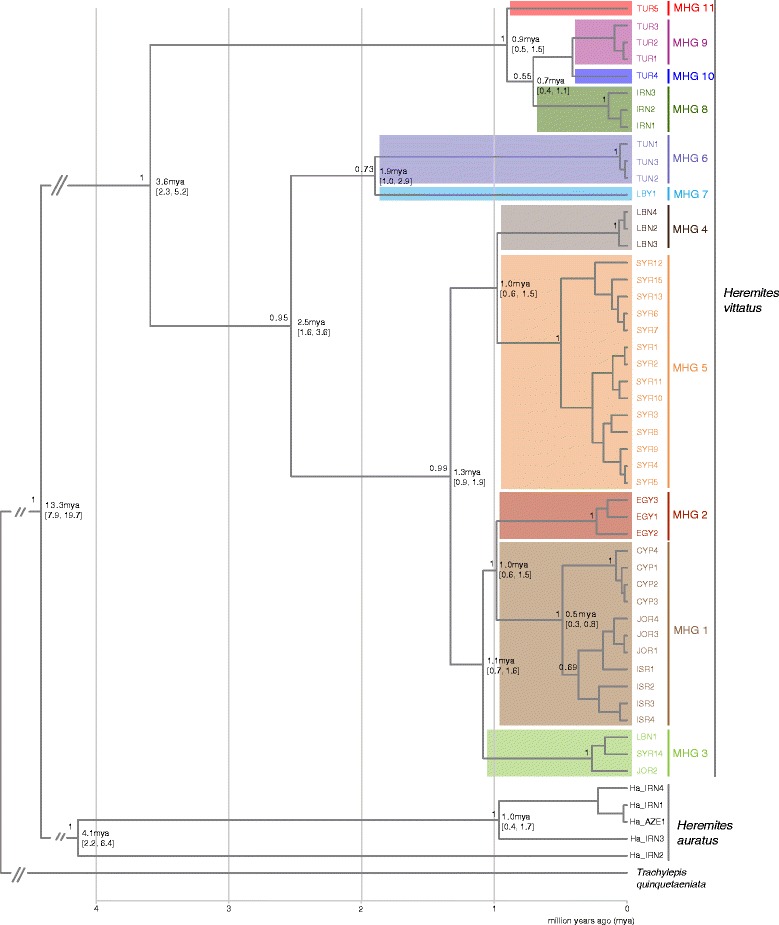
Bayesian inference tree of *Heremites vittatus*. Numbers above branches are Bayesian posterior probabilities; only values >0.5 are shown. Numbers at nodes are divergence times in million years ago (mya), with the 95% HPD interval in parentheses

**Fig. 5:**
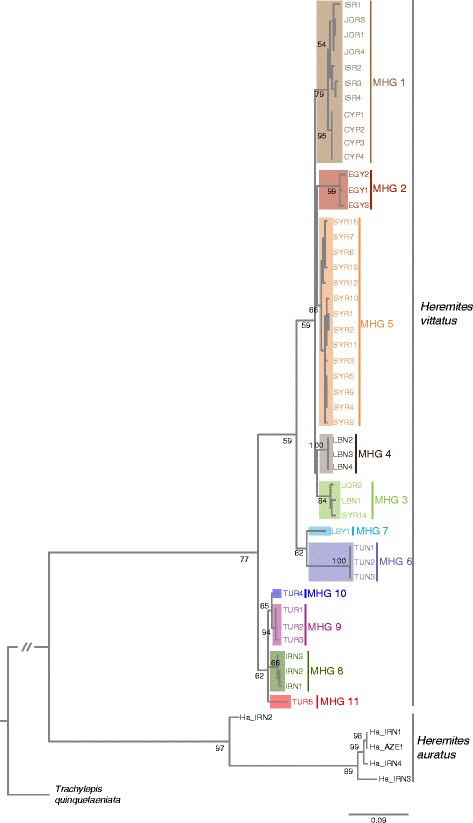
Maximum likelihood tree of *Heremites vittatus*. The percentage of trees in which the associated taxa clustered together is shown next to the branches; only values >50 are shown. The tree is drawn to scale, with branch lengths measured in the number of substitutions per site

Our mitochondrial phylogeography supported five key findings (Figs. 3, 4, 5; Additional file 2). (1) Both *Heremites auratus* and *Heremites vittatus* formed well-supported lineages that diverged 13.3 million years ago (mya), and were separated by a mean *p*-distance of 0.189 ± 0.017. (2) Inside *H. vittatus*, two highly supported lineages at the periphery of the distribution range branched off early. The Turkish/Iranian lineage (MHGs 8–11) branched off first 3.6 mya (mean *p*-distance to eastern Mediterranean lineage: 0.090 ± 0.012; mean *p*-distance to Tunisian/Libyan lineage: 0.101 ± 0.013). The Tunisian/Libyan lineage (MHGs 6–7) diverged 2.5 mya (*p*-distance to the eastern Mediterranean lineage: 0.092 ± 0.011); within this lineage, the Tunisian samples (MHG 6) and the Libyan sample (MHG 7) diverged 1.9 mya (*p*-distance: 0.079 ± 0.013). (3) In the BEAST analysis, the eastern Mediterranean lineage bifurcated into a northern (MHG 4: Lebanon; MHG 5: Syria) and a southern lineage (MHG 1: Cyprus, Jordan, Israel; MHG 2: Egypt; MHG 3: Jordan, Lebanon, southern Syria) approximately 1.3 mya (*p*-distance: 0.039 ± 0.007). (4) The samples from Cyprus constituted a monophyletic group that diverged from the Israeli/Jordanian lineage ca. 0.5 mya (*p*-distance: 0.015 ± 0.005). (5) *H. auratus* comprised two deeply diverged lineages: The sample from northeastern Iran (Ha_IRN2) was sister to the samples from western Iran (*p*-distance: 0.122 ± 0.015); these two lineages diverged 4.1 mya. Compared to the samples from western Iran, the Ha_IRN2 sample had a short branch length in the RAxML tree.

### Morphological analysis

**Fig. 6:**
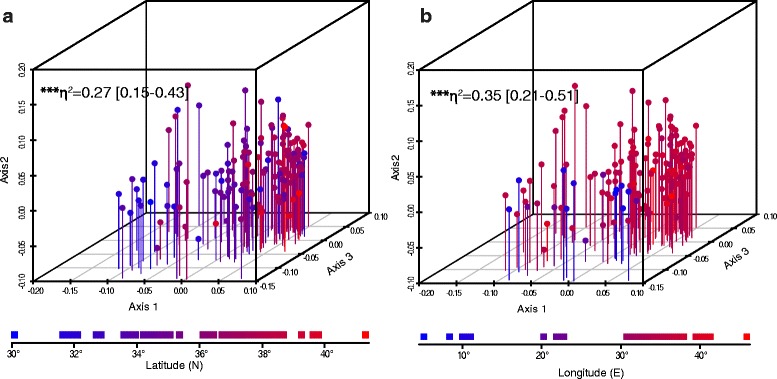
Geographical patterns of multivariate morphological variation in *Heremites vittatus*. 3D projection of the first, second and third axis of the multidimensional scaling analysis (MDS) of 27 morphological traits (*n* = 146 specimens). MDS axes 2 and 3 are plotted in Z and Y planes, respectively, for easier demonstration of spatial patterns. Samples are color-coded to indicate latitude (**a**) or longitude (**b**) of the collection site, according to the color gradient legends at the bottom. η^2^ estimates represent the effect of latitude/longitude on the position in the morphospace (point estimate: type 2 MANOVA using Pillai’s test on results of multivariate multiple regression; 95% confidence intervals: Bootstrap analysis with 10^5^ repeats). In all figures, NS = not significant, * *P* < 0.05, ** *P* < 0.01, *** *P* < 0.001, **** *P* < 0.0001

We first performed a non-metric multidimensional scaling (NMS) analysis to assess the morphological variation in an unbiased way. A 3D plot of the first three NMS axes did not reveal any discrete grouping of samples in the morphospace (Fig. 6). To test for an effect of geographical location on the position in the morphospace, we color-coded samples according to their latitude (Fig. 6a) and longitude (Fig. 6b). Interestingly, both latitude and longitude had significant effects on the position of samples in the morphospace (multivariate multiple regression; latitude: η^2^ = 0.27 [0.15–0.43], F = 17.23, *P* = 1.4e-9; longitude: η^2^ = 0.35 [0.21–0.51], F = 25.74, *P* = 2.45e-13), such that samples revealed a southwest–northeast differentiation.

**Fig. 7:**
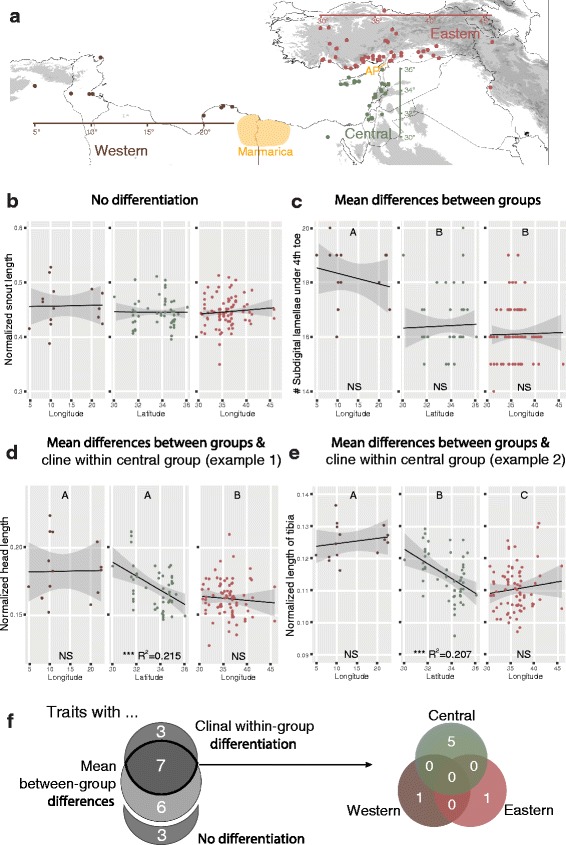
Clinal vs. mean trait differentiation in *Heremites vittatus*. **a** Map of sample locations and assignment to geographical groups (western, central, eastern). Dispersal barriers (Marmarica region, Amik Plain [AP]) that delimit the geographical groups are indicated in orange. Traits were first tested for significant differences between groups (Dunnett’s modified Tukey-Kramer [DTK] test). Different letters above graphs indicate significant mean differences between groups. For analysis of clinal variation, groups were then regressed against longitude (western, eastern) or latitude (central). Multiple R^2^ values and significance of regression are indicated below graphs. **b** Example of a trait that does not show mean differences between groups or clinal variation within groups. **c** Example of a trait that shows mean differences between groups, but no evidence of clinal variation within groups. **d**, **e** Examples of traits that show mean differences between groups, and clinal variation in the central group. **f** Summary of patterns of variation in the 19 analyzed traits

These results could be explained by morphological divergence between the three main mitochondrial lineages (Tunisian/Libyan, Turkish/Iranian, eastern Mediterranean) in our phylogeographic analysis, because these lineages are distributed in an allopatric, roughly longitudinal way. To examine this hypothesis in our morphological dataset, we assigned specimens based on their collection localities to three morphological groups (western, central, eastern) coinciding with the putative ranges of the three mitochondrial lineages (Fig. 7a). Their ranges are delimited by prominent barriers for animal dispersal: The Tunisian/Libyan lineage is separated from the eastern Mediterranean lineage by the arid Marmarica region (located between the Cyrenaica and the Nile delta) [41], while the eastern Mediterranean lineage is separated from the Turkish/Iranian lineage by the Amik plain in southern Turkey (Fig. 7a) [42, 43]. We then tested traits individually for significant mean differences between these groups. We excluded binary and meristic traits that showed little variation and are unlikely to contribute to the pattern of variation, and focussed on five meristic and 14 mensural traits. Consistent with the hypothesis of morphological divergence between the mitochondrial lineages, we found that the majority of traits showed significant mean differences between the western, central, and eastern group (13/19 traits, 68%; Table 2, Fig. 7f).

**Table 2:** Within-group mean and range of traits with significant mean differences between groups. Trait differences between groups were tested for significance with Dunnett’s modified Tukey-Kramer (DTK) test

	Western (W)	Central (C)	Eastern (E)	Significance
Trait	mean (min-max)	mean (min-max)	mean (min-max)	W-E; W-C; C-E
# middorsals (MDN)	54.73 (52–57)	55.16 (52–59)	56.7 (52–61)	**; NS; ****
# midventrals (MVN)	36.93 (34–40)	41.72 (36–48)	40.93 (36–46)	****; ****; NS
# longitudinal scale rows (LSN)	33.27 (32–36)	32.5 (31–34)	31.68 (30–34)	***; NS; ****
# subdigital lamellae under 4th toe (SDLN)	18.2 (16–20)	16.41 (15–20)	16.1 (14–19)	****; ****; NS
Normalized width of tail at tail base (HTc)	0.070 (0.050–0.097)	0.090 (0.042–0.130)	0.099 (0.039–0.128)	****; **; NS
Normalized height of tail at tail base (VTc)	0.069 (0.040–0.107)	0.090 (0.035–0.127)	0.098 (0.039–0.127)	***; **; NS
Normalized head length (HLc)	0.183 (0.152–0.224)	0.171 (0.147–0.212)	0.162 (0.128–0.210)	**; NS; *
Normalized head width (HBc)	0.729 (0.647–0.824)	0.749 (0.604–0.924)	0.767 (0.667–0.948)	*; NS; NS
Normalized length of hind leg (LHc)	0.355 (0.328–0.381)	0.348 (0.285–0.403)	0.334 (0.287–0.388)	**; NS; *
Normalized length of front leg (LFc)	0.229 (0.199–0.270)	0.232 (0.184–0.281)	0.220 (0.166–0.265)	NS; NS; *
Normalized length of tibia (LTc)	0.125 (0.116–0.137)	0.115 (0.096–0.129)	0.111 (0.097–0.131)	****; ****; **
Normalized length of forearm (LAc)	0.091 (0.082–0.105)	0.085 (0.074–0.098)	0.081 (0.069–0.101)	***; **; *
Normalized inter-nare distance (INAc)	0.170 (0.117–0.214)	0.179 (0.127–0.229)	0.190 (0.152–0.235)	*; NS; *

Alternatively, the effect of latitude/longitude on the position in the morphospace could be explained by clinal variation within geographical groups. While the western (Algeria, Tunisia, Libya) and eastern (Turkey, Iran) groups have a roughly longitudinal distribution, the central group (Egypt, Israel, Jordan, Lebanon, Cyprus, Syria) is predominantly distributed along the latitudinal dimension (Fig. 7a). To account for these different geographical extensions, we regressed traits against longitude for the western and eastern group, and against latitude for the central group. 10 of 19 traits (53%) showed evidence of clinal variation in one geographical group. No trait showed evidence for clinal variation in more than one group. Of the 10 traits, seven showed clinal variation in the central group. Two traits showed clinal variation in the western group, and one trait in the eastern group (Fig. 7f).

We next investigated how mean differences between groups overlap with clinal variation within groups. Of 19 traits, three (16%) showed evidence of neither mean nor clinal variation (Fig. 7b). Six traits (32%) showed evidence of only mean differences (Fig. 7c), while three traits (16%) showed evidence of only clinal variation. Seven traits (37%) showed evidence of both mean and clinal variation. Of the latter seven traits, five showed clinal variation in the central group (Fig. 7d-e). Taken together, our results suggest that the majority of morphological traits in *H. vittatus* exhibit mean differences between geographical groups with either no evidence for clinal variation or clinal variation along the eastern Mediterranean coast.

## Discussion

### Historical biogeography

Our mitochondrial phylogeography suggests that *Heremites vittatus* diverged from *H. auratus* in the late Miocene (13.3 mya; range 7.9–19.7 mya), consistent with a previous estimate that dated this split to 10.54 mya [44]. *Heremites vittatus* likely emerged as a distinct species in southwest Asia, where the *Heremites* radiation originated [23]. In the mid Pliocene (3.6 mya; range 2.3–5.2 mya), the ancestors of the eastern Mediterranean and northern African lineages split off from the ancestral Irano-Anatolian lineage and became allopatric. This divergence event coincided with prominent geological events in Anatolia during the late Neogene. The Anatolian mountain ranges, e.g., the Amanus and Taurus mountains, created geographical barriers between the Anatolian and eastern Mediterranean regions in the South. The vast lake system in central Anatolia delimited the distribution into western Anatolia. Other emerging dispersal barriers subsequently supported this divergence, notably the Amik plain in southern Turkey. As a result, vicariant cladogenesis, frequent among other animals in this geologically complex region (e.g. green lizards), may explain the divergence of the Irano-Anatolian lineage and the ancestors of the eastern Mediterranean and northern African lineages [14, 43, 45, 46].

During much of the Pliocene, the climate was relatively warm and humid in the Palearctic [47], thus potentially facilitating the colonization of northern Africa from the eastern Mediterranean. In the late Pliocene, however, the climate suddenly aridified and became colder [41, 48, 49]. Around this time, the northern African lineages split off (2.5 mya; range 1.6–3.6 mya). Consistent with the preference of the modern species for more humid habitats in northern Africa [24, 37], this climatic turn may have restricted the species to the remaining humid regions, and isolated them from populations inhabiting the eastern Mediterranean coast. Similar divergence events coinciding with the transition from hotter/wetter to colder/drier climate in the late Pliocene/early Pleistocene have previously been observed in other animal species of the Mediterranean (e.g., tree frogs, *Typhlops vermicularis*) [8, 11, 50]. Notably, *H. vittatus* is the only *Heremites* species in northern Africa today, suggesting that other *Heremites* species never migrated into northern Africa, or were unable to withstand the drastic climatic changes at the Pliocene/Pleistocene transition.

During the Pleistocene, the Sahara increasingly aridified, which restricted more humid habitats to a few coastal areas (e.g., Cyrenaica) and oases [41, 51]. This aridification may have led to the further fragmentation of the northern African lineage, as evidenced by the deep split between the Tunisian and Libyan haplotypes in the Cyrenaica (1.9 mya; range 1.0–2.9 mya). Although these climatic cycles must have influenced distributions during the Pleistocene, the mitochondrial lineages remained separate and apparently did not mix. The extant, allopatric populations in Libya, Tunisia, and Algeria are thus potential remnants of a once more extended distribution in northern Africa. Similar fragmentation processes in the Pleistocene likely affected other animal species in northern Africa currently limited to the Cyrenaica and coastal areas of Tunisia and Algeria, such as lizards of the genus *Ophisops* [12].

In the late Pleistocene, the Irano-Anatolian and eastern Mediterranean lineages further radiated, establishing the mitochondrial diversity observed today. The Egyptian, Cypriot-Israeli-Jordanian, Jordanian-Lebanese-Syrian, Lebanese, and Syrian major haplogroups (MHGs) all evolved into monophyletic lineages during this time. Based on our dataset, most of these lineages occupy non-overlapping regions, suggesting that these haplotypes are not admixed. One interesting exception is the co-occurrence of the Jordanian-Lebanese-Syrian MHG with the Cypriot-Israeli-Jordanian MHG at one locality in Jordan (Al Himma), raising the possibility of admixture between these lineages in this region. In our dataset, the monophyletic lineage on the island of Cyprus is sister to the lineages in Israel and Jordan and diverged approximately 0.5 mya (range 0.3–0.8 mya). During the Pleistocene, Cyprus was likely never connected to the mainland through a land bridge [25, 52], so that the species must have reached Cyprus via overseas dispersal, which has also been suggested for other species in Cyprus, e.g., *Hyla savignyi* [8, 25, 53]. However, these hypotheses may not be correct if the true mainland source of the Cypriot population was not included in our dataset.

Although our study shows again the usefulness of fast-evolving mtDNA genes to dissect more recent intraspecific divergence in Palearctic reptiles (e.g., [54–58]), we cannot rule out the possibility of discordant evolutionary histories of the mitochondrial and nuclear genome [59–61]. The mtDNA lineage evolution described in this study should thus be corroborated with nuclear sequence variation before additional inferences (e.g. for taxonomic purposes) are made on the evolutionary history of this complex.

### Morphological evolution

Our multidimensional scaling analysis suggests that morphological variation in *Heremites vittatus* is linked to latitude and longitude. At the level of individual traits, most show mean differences between three morphological groups (western, central, eastern) coinciding with the putative ranges of the three old mitochondrial lineages. We note that while these results are suggestive, proof of concordant genetic and morphological variation would require data collection from identical specimens. Interestingly, we further find that these traits show either (1) no evidence for within-group clines, or (2) clinal latitudinal variation in the central (eastern Mediterranean) group.

The first category, mean differences with no evidence for within-group clines, argues for a close association of trait variation with the three old mtDNA lineages. For example, the western (northern African) group has an average of 18.2 subdigital lamellae, while the central (eastern Mediterranean) and eastern (Turkish/Iranian) group have on average 16.4 and 16.1, respectively, with no evidence for a clinal increase of lamellae in the central group (Fig. 7c). Thus, although they are likely not closely related the eastern and central group share a similar number of subdigital lamellae, which may reflect an ancestral state that was independently maintained in both lineages. By contrast, the higher number of subdigital lamellae in the western group may be an adaptation to the xeric environmental conditions across northern Africa because sand-dwelling lizards often possess pedal specializations that evolved as (convergent) adaptations to sandy habitats [62]. Mean differences between geographical groups can thus be explained by lineage-specific ancestral or derived trait states.

The second category, mean differences between groups with latitudinal clines within the central (eastern Mediterranean) group, suggests that some traits are affected by selection gradients in addition to lineage-specific trait states. One such trait is relative head length. Lizards in the western group have on average longer heads relative to their body length than the eastern group, with no evidence of clinal variation in either group. By contrast, relative head length in the geographically intermediate central group decreases gradually from the western (northern African) to eastern (Turkish/Iranian) trait states: the farther north specimens were collected along the eastern Mediterranean coast, the shorter their heads are relative to body length. The large extension (roughly 1000 km) of this cline suggests that the central group is likely not a hybridization zone of the western and eastern group, but rather a distinct population affected by gradual environmental change. Remarkably, we found little evidence that similar clines exist in the western or eastern group, pointing to unique environmental changes at the transition from northern Africa to Anatolia [45, 46].

Taken together, our morphological analysis reveals that morphological variation in *Heremites vittatus* has a clear geographical organization, and is likely both affected by lineage-specific trait conservation and local trait adaptation.

## Conclusions

Many species in the Middle East and northern Africa exhibit predominantly longitudinal distributions, and are potentially characterized by longitudinal patterns of biological diversity, but the underlying biogeographic mechanisms and evolutionary timescales remain largely unclear. In this paper, we provide the first comprehensive assessment of the mitochondrial phylogeography and multivariate morphological variation of the striped skink, *Heremites vittatus*, a species with a predominantly longitudinal distribution across the Middle East and northern Africa. We suggest that *H. vittatus* evolved as a separate species in southwest Asia in the late Miocene. We show that the species diverged into three main mitochondrial lineages roughly 2.5–3.6 mya in the eastern, central, and western regions of the distribution range, introducing a longitudinal organization of genetic diversity. This divergence was likely driven by a combination of geological changes in Anatolia and climatic changes in northern Africa. The morphological variation reflects this pattern of mitochondrial divergence: the majority of morphological traits show significant differences between these regions, supporting the idea of independently evolving lineages. We also find that subsequent local radiation in the Pleistocene, likely driven by climatic oscillations and locally emerging geographical barriers, led to the mitochondrial diversity observed today. Morphologically, clinal variation along the eastern Mediterranean coast in a subset of traits suggests that ongoing selection pressures and local adaptation may play an important role in shaping populations at the transition from northern Africa to Anatolia. Our findings of allopatric, morphologically and genetically divergent lineages raise the possibility that *Heremites vittatus* represents a complex with several undescribed taxa. However, taxonomic decisions should be based on nuclear, mitochondrial, and morphological data collected in the context of future studies on the inter- and intraspecific systematics and taxonomy of *Heremites*. In conclusion, our study suggests that longitudinal patterns and species distributions in the Middle East and northern Africa may be the result of complex evolutionary processes, driven by the region’s geological and climatic history, geographical setup, and the presence of the Sahara.

## Methods

### DNA sequence analysis

The DNA sampling consists of 46 *H. vittatus* specimens (Additional file 3), including one sequence from GenBank, covering most of the distribution range (Fig. 1a). We also sequenced five *H. auratus* specimens*.* We included *Trachylepis quinquetaeniata* from the African lineage of *Mabuya* s.l. as an outgroup, because this lineage is one of the closest relatives of Middle Eastern *Heremites* [23, 63–65]. We did not include in the analyses sequences of *H. vittatus* from GenBank that only partially overlap the alignment generated in this study (but see Additional file 2).

Small pieces of tissue were sampled either from museum specimens (toe or tongue) of various ages and storage conditions, or were provided by colleagues based on field collections (part of tail or liver). Samples were stored in 70% ethanol or EDTA buffer, and genomic DNA was extracted with standard protocols [66]. A portion of the mitochondrial *cytochrome b* gene was amplified by polymerase chain reaction (PCR) with the primers mt-c-emys (5′-CCG GAT CAA ACA AYC CAA CAG G-3′) [67] and mt-fs-h (5′-CCA GTA GAA CAC CCA TTC ATC ATC ATT GGC CAA CTA-3′) [68]. This resulted in an amplicon with a length of 394 bp, corresponding to positions 14,762–15,155 in the mitochondrial genome of *Eumeces egregius* (positions 633–1026 in the *cytochrome b* gene of *E. egregius*) [69].

DNA sequences were aligned with the ClustalW algorithm implemented in BioEdit [70], and SNPs were verified by checking the sequence chromatograms. No insertions or deletions were found in the alignment. We deposited new sequences in GenBank under accession numbers MF101923-MF101972 (see Additional file 3). We used MEGA5.2.2 [71] to determine nucleotide diversity and to calculate uncorrected p-distances based on pairwise deletion of ambiguous sites, and DnaSP [72] to identify haplotypes and determine haplotype diversity. We then divided the dataset by codon position, and ran PartitionFinder 1.1.1 [73] to find the best partition scheme and associated substitution models. The best partition scheme included the three codon positions each as a separate partition (lnL = −1930.14357, BIC = 4553.24905498), with the following models: K80 + G (position 1), HKY + I (position 2), TrN + G (position 3).

We used BEAST v1.8.1 and associated software tools [74] to obtain time-calibrated estimates of phylogenetic divergence. We used the partitioned dataset, and set the substitution models available in BEAUTi equivalent to the PartitionFinder model selection for the partitions separately (partition 1: HKY with gamma site heterogeneity; partition 2: HKY with invariant site heterogeneity; partition 3: TN93 with gamma site heterogeneity). In accordance with previous recommendations [75], we used an uncorrelated lognormal relaxed clock model with a constant coalescent tree prior and a random starting tree for all three partitions. Since dated fossils are not available for any *Heremites* species or related genera (*Trachylepis, Chioninia*, *Eutropis*, *Mabuya* sensu stricto), we instead generated time-calibrated divergence times through estimates of the substitution rate. Substitution rates for *cytochrome b* in other skinks (*Eumeces, Chalcides, Scincus*), geckos (*Hemidactylus*), and lacertid lizards (Lacertini) range from 1.15–1.35% per million years [64, 76–78]. We used a normally distributed prior of the substitution rate (ucld) with an initial mean of 1.25% and a standard deviation of 0.5% for all partitions in BEAUTi, and then optimized these values by checking for convergence and high ESS scores in Tracer v1.6. We then ran five separate rounds of each 10^7^ MCMC iterations and logged parameters every 1000 steps, which generated 50,000 trees. We combined these runs in LogCombiner v1.8.1 with a burn-in of 10% of each run, generated a consensus tree with TreeAnnotator v1.8.1, and produced the final tree with FigTree v1.4.2.

In addition, we calculated a maximum likelihood (ML) tree with the program RAxML 7.0.4 [79] using the rapid hill climbing algorithm [80]. The dataset was partitioned into the three codon positions corresponding to the PartitionFinder partitioning strategy, and run under the suggested [79] GTR + G substitution model in RAxML.

Sequence evolution within a species does not necessarily follow dichotomous patterns, because co-existence of ancestral and derived haplotypes may introduce reticulate patterns of evolutionary relationships. To account for this phenomenon, we computed a haplotype network with the software NETWORK v4.611 [81] (http://www.fluxus-engineering.com), using the median-joining (MJ) algorithm with default settings (epsilon = 0). We assigned haplotypes to major haplogroups (MHGs) with the Bayesian implementation of the Poisson tree processes (PTP) model [82], a method to delimit putative species on a phylogenetic tree, using the BEAST tree (Fig. 4) and the PTP default settings (100,000 MCMC generations, thinning 100, burn-in 0.1, seed 123).

### Morphological analysis

We examined 146 *H. vittatus* specimens from across the distribution range of the species (Additional file 4, Fig. 1b). Specimens were only included in the dataset if the collection locality could be reliably geo-referenced. We did not distinguish among sexes of the examined specimens. For bilateral traits, the left character state was recorded. We measured 60 traits and excluded traits that showed no or only sporadic variation, leaving two binary, eleven meristic and 14 mensural traits for further analyses (Additional file 4). All mensural characters, except for snout-vent length, were first normalized for size by dividing through the snout-vent length (TL, HT, VT, ALL, HL, TH, LH, LF, LT, LA) or the head length (SL, HB, INA), respectively. Data were then ln(x + 1)-transformed. We first conducted a non-metric multidimensional scaling analysis (NMS), which is a statistical approach to reduce the dimensionality of the data set by estimating the similarity of every pairwise comparison of samples based on a similarity coefficient. We used the Gower index (= range-normalized Manhattan index) for meristic and mensural traits, and the Jaccard index for binary characters as defaulted in the program PAST [83]. We retrieved the first three NMS axes and performed a multivariate multiple regression to test for the effect of geographical location on the 3D position in morphospace. We summarized the results with a type 2 MANOVA using Pillai’s test to obtain separate η^2^ point estimates for the effect of latitude and longitude, and conducted a Bootstrap analysis with 10^5^ repeats to obtain 95% confidence intervals for the η^2^ point estimates. We restricted subsequent analyses to five meristic and 14 mensural traits that showed substantial variation, and assigned specimens to three geographical groups (western, central, eastern). We conducted pairwise multiple comparison tests for significant mean differences between these groups using Dunnett’s modified Tukey-Kramer test for uneven sample sizes and heterogeneous variance as implemented in the “DTK” R package. We also regressed individual traits separately for each group against the longitude (western and eastern groups) and latitude (central group). All statistical analyses were done in R software v3.2.4 [84].

## Additional files

Additional file 1: PTP tree used to delimit major haplogroups. Values on nodes are Bayesian support (BS) values. Higher BS values indicate that descendants from this node are more likely to be from one species. Nodes and their descendant branches that were assigned to the same species by PTP are red; singleton species are left in blue. (PDF 248 kb)

Additional file 2: Maximum Likelihood (ML) tree based on combined sequences of this study and *cytochrome b* sequences of *Heremites vittatus* from Turkey (in green, retrieved from GenBank [44]) that partially overlap (positions 1-187) with the *cytochrome b* alignment (394bp) presented here. The tree was calculated with RAxML 7.0.4 using the climbing hill algorithm. The dataset was partitioned into the three codon positions, and run with a GTR+G substitution model in RAxML. (PDF 127 kb)

Additional file 3: Samples included in the phylogeographic analysis. FMNH = Field Museum of Natural History, Chicago, USA; HUJR = Hebrew University Jerusalem, Israel; IPMB = Tissue sample collection, Institute for Pharmacy and Molecular Biotechnology, University of Heidelberg, Germany; NHMC = Natural History Museum of Crete, University of Crete, Heraklion, Greece; NMW = Natural History Museum Vienna, Austria; ZFMK = Zoological Research Museum A. Koenig, Bonn, Germany; ZSM = Zoological State Collection, Munich, Germany. (XLSX 15 kb)

Additional file 4: Voucher specimens and raw data included in the morphological analysis. Country codes: CYP = Cyprus, DZA = Algeria, EGY = Egypt, IRN = Iran, ISR = Israel, JOR = Jordan, LBN = Lebanon, LBY = Libya, SYR = Syria, TUN = Tunisia, TUR = Turkey. Group codes: W = Western, C = Central, E = Eastern. Morphological trait abbreviations, definitions, and data types: PF (prefrontals in contact, binary), P (parietals in contact, binary), NRN (# scales per row of nuchals, meristic), ILN (# infralabials, meristic), ISC (# infralabials in contact with subocular, meristic), SCN (# supraciliaries, meristic), ICF (which infralabial does the first chin scale reach? meristic), ICS (which infralabial does the second chin scale reach? meristic), MDN (# middorsals, from the nuchals to the middle of the hind leg base, meristic), MVN (# midventrals, from the axilla to the hind leg base, meristic), LSN (# longitudinal scale rows around midbody, meristic), SDLN (# subdigital lamellae, meristic), FL (# lamellae under fourth finger, meristic), SVL (snout-vent length, mensural), TL (tail length, mensural), HT (width of tail at tail base, mensural), VT (height of tail at tail base, mensural), ALL (distance from axilla to anterior base of hind legs, mensural), HL (head length, from tip of snout to anterior end of ear, mensural), SL (snout length, tip of snout to anterior end of eye, mensural), HB (head width, at greatest width of head, mensural), TH (thoracic height, at shoulder, mensural), LH (length of hind leg, mensural), LF (length of front leg, mensural), LT (length of tibia, mensural), LA (length of forearm, mensural), INA (inter-nare distance, mensural). Abbreviations of museum collections are the same as in Additional file 3, in addition to: SMF = Senckenberg Museum Frankfurt, Germany; MHNG = Natural History Museum of Geneva, Switzerland; MTD = Senckenberg Museum of Zoology, Dresden, Germany. (XLSX 41 kb)
