# Supplementary figures and images for: Pre-Quaternary divergence and subsequent radiation explain longitudinal patterns of genetic and morphological variation in the striped skink, Heremites vittatus
Source: BMC Evol Biol. 2017 Jun 9;17:132. doi: 10.1186/s12862-017-0969-0 (PMC5466720; doi:10.1186/s12862-017-0969-0)

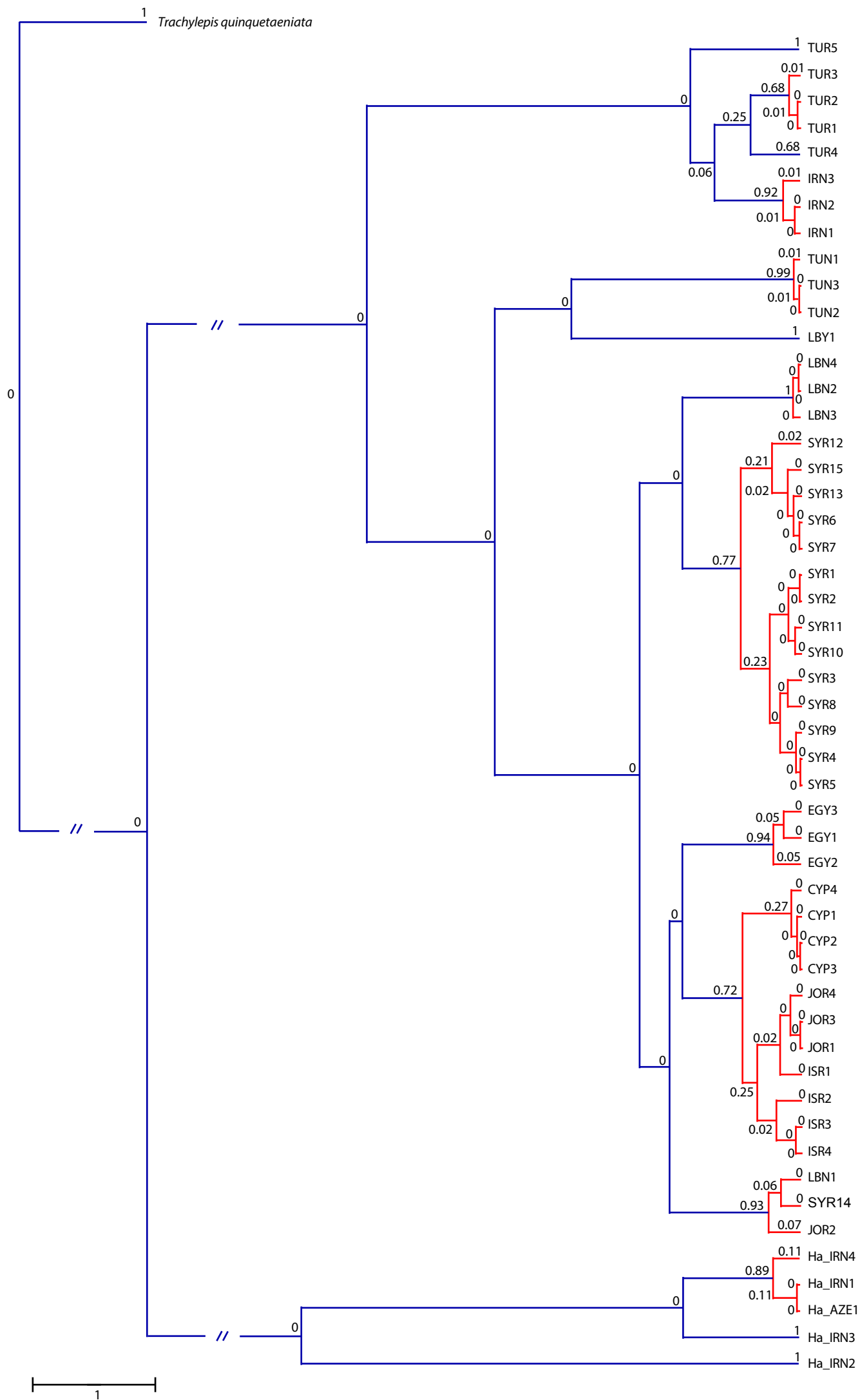

Supplement: Supplementary file 1 — PTP tree used to delimit major haplogroups. Values on nodes are Bayesian support (BS) values. Higher BS values indicate that descendants from this node are more likely to be from one species. Nodes and their descendant branches that were assigned to the same species by PTP are red; singleton species are left in blue. (PDF 248 kb) [file 12862_2017_969_MOESM1_ESM.pdf]
