# Supplementary material for: Pre-Quaternary divergence and subsequent radiation explain longitudinal patterns of genetic and morphological variation in the striped skink, Heremites vittatus
Source: BMC Evol Biol. 2017 Jun 9;17:132. doi: 10.1186/s12862-017-0969-0 (PMC5466720; doi:10.1186/s12862-017-0969-0)
Supplement: Supplementary file 2 — Maximum Likelihood (ML) tree based on combined sequences of this study and cytochrome b sequences of Heremites vittatus from Turkey (in green, retrieved from GenBank [44]) that partially overlap (positions 1-187) with the cytochrome b alignment (394bp) presented here. The tree was calculated with RAxML 7.0.4 using the climbing hill algorithm. The dataset was partitioned into the three codon positions, and run with a GTR+G substitution model in RAxML. (PDF 127 kb) [file 12862_2017_969_MOESM2_ESM.pdf]

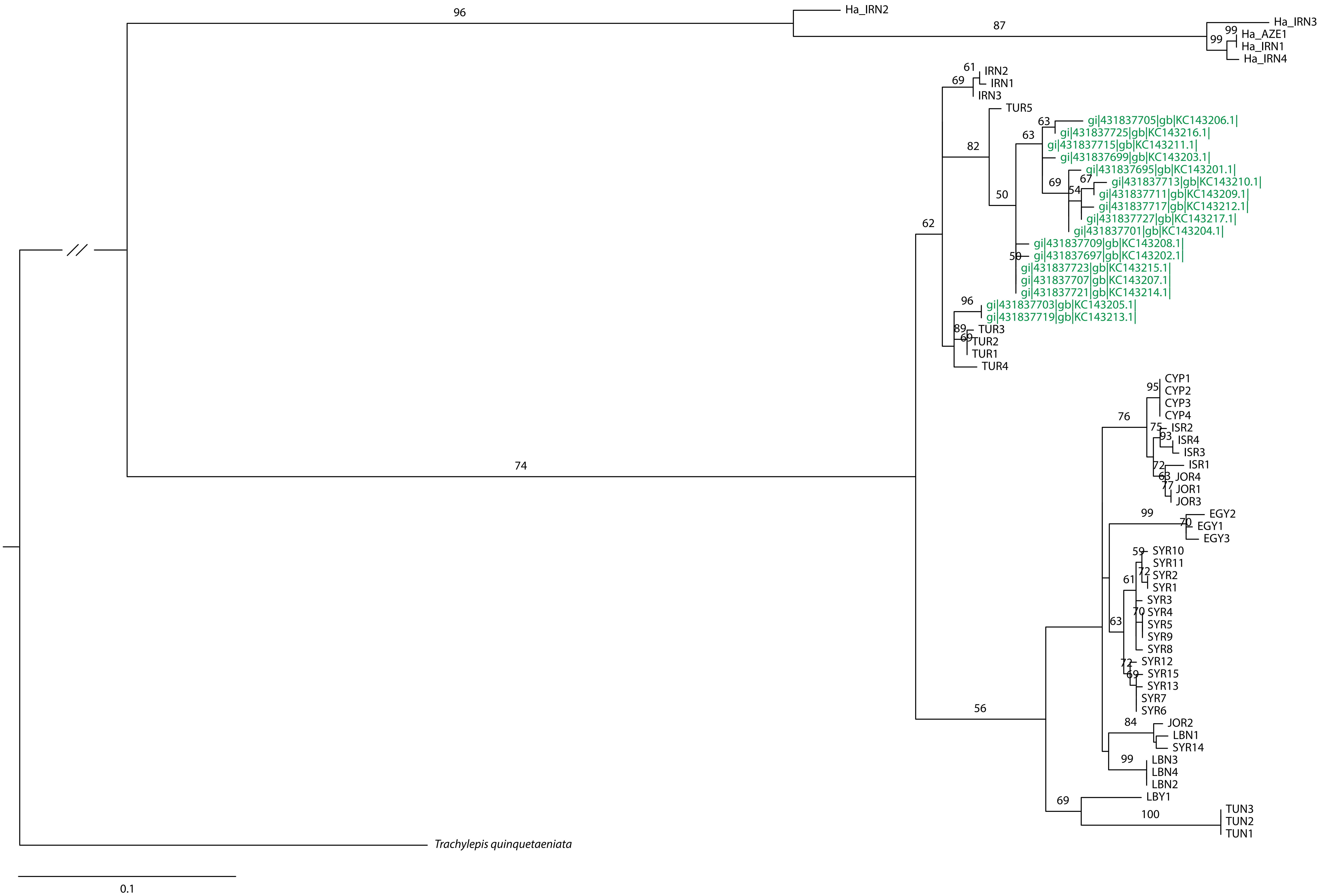

Additional file 2: Maximum Likelihood (ML) tree based on combined sequences of this study and *cytochrome b* sequences of *Heremites vittatus* from Turkey (in green, retrieved from GenBank [ref. 44]) that partially overlap (positions 1-187) with the *cytochrome b* alignment (394bp) presented here. The tree was calculated with RAxML 7.0.4 using the climbing hill algorithm. The dataset was partitioned into the three codon positions, and run with a GTR+G substitution model in RAxML.
